# Health Impacts of Exposure to Gaseous Pollutants and Particulate Matter in Beijing—A Non-Linear Analysis Based on the New Evidence

**DOI:** 10.3390/ijerph15091969

**Published:** 2018-09-10

**Authors:** Yunfei Cheng, Tatiana Ermolieva, Gui-Ying Cao, Xiaoying Zheng

**Affiliations:** 1 Institution of Population Research, Peking University, Beijing 100871, China; chengyunfei@pku.edu.cn; 2 Exploratory and Special Projects, International Institution for Applied Systems Analysis, Laxenburg 2361, Austria; cao@iiasa.ac.at; 3 Ecosystems Services and Management, International Institution for Applied Systems Analysis, Laxenburg 2361, Austria; ermol@iiasa.ac.at

**Keywords:** air pollution control, gaseous pollutants, respiratory diseases

## Abstract

This paper aimed to estimate health risks focusing on respiratory diseases from exposure to gaseous multi-pollutants based on new data and revealed new evidence after the most stringent air pollution control plan in Beijing which was carried out in 2013. It used daily respiratory diseases outpatient data from a hospital located in Beijing with daily meteorological data and monitor data of air pollutants from local authorities. All data were collected from 2014 to 2016. Distributed lag non-linear model was employed. Results indicated that NO_2_ and CO had positive association with outpatients number on the day of the exposure (1.045 (95% confidence interval (CI): 1.003, 1.089) for CO and 1.022 (95% CI: 1.008, 1.036) for NO_2_) (and on the day after the exposure (1.026 (95% CI: 1.005, 1.048) for CO and 1.013 (95% CI: 1.005, 1.021) for NO_2_). Relative risk (RR) generally declines with the number of lags; ozone produces significant effects on the first day (RR = 0.993 (95% CI: 0.989, 0.998)) as well as second day (RR = 0.995 (95% CI: 0.991, 0.999)) after the exposure, while particulate pollutants did not produce significant effects. Effects from the short-term exposure to gaseous pollutants were robust after controlling for particulate matters. Our results contribute to a comprehensive understanding of the dependencies between the change of air pollutants concentration and their health effects in Beijing after the implementation of promising air regulations in 2013. Results of the study can be used to develop relevant measures minimizing the adverse health consequences of air pollutants and supporting sustainable development of Beijing as well as other rapidly growing Asian cities.

## 1. Introduction

In recent years, China has experienced severe air pollution problems [1,2,3,4]. Satellite and ground observations show that the concentrations of particulate matter and gaseous pollutants have become extremely high in northern China over recent decades [5,6,7]. Located in the highly developed area of northern China, Beijing has a serious air pollution problem and public health is threatened [2,5,8,9].

To respond to the raising public health concerns and air pollution, “Action Plan for Air Pollution Control” was introduced in September 2013. It required Beijing-Tianjin-Hebei (Jing-Jin-Ji) area to reduce its concentration of PM_2.5_ by 25% in 2017 compared to its 2013 levels, and the annual concentration of PM_2.5_ in Beijing should be kept around 60 µg/m^3^ [10]. The detailed plans have included closing unqualified industries, restructuring and relocating factories [11]. 

Meanwhile, the number of motor vehicles in Beijing has increased dramatically from 4.98 million in 2011 [12] to 5.71 million in 2016 [13]. The change of industry structure and relocation, accompanied by extensive use of vehicles, made vehicle exhaust a major pollutant source in Beijing [14,15]. 

Outdoor air pollutants are generally divided into gaseous pollutant (e.g., SO_2_, NO_2_, CO, O_3_) and particulate matter (e.g., PM_2.5_, PM_10_) [16]. Publications have revealed evidence that air pollutants may trigger pulmonary oxidative stress and inflammation [17,18,19,20] and most of existing studies consistently show that particulate matter has significant associations with adverse health effects [21,22,23,24,25,26,27]. However, results regarding exposure to gaseous pollutants have been less conclusive [28,29].

Evidence from epidemiological studies of air pollution have played a vital role in policymaking and standard-setting [30]. Air quality health impact assessment and standards revision in developing countries have mainly relied on the extrapolation of results from epidemiological studies obtained from Europe and North America [31]. The health effects of particulate matter have been comparatively less investigated, especially for Asian countries, despite the fact that these countries typically have much higher levels of exposure [32]. This has raised several uncertainties as disagreement remained about to what extent coefficients of exposure-response relationships varied under different levels of air pollution [33,34,35]. 

Furthermore, most published studies relating to Beijing used data collected before 2013, which failed to reflect the trend and circumstances after the implementation of “Action Plan for Air Pollution Control” since 2013. For the next stage of air pollution improvement strategies and public health policies, there is a need to assess the effects of air pollution on heath based on new evidence and the latest observed data. 

In this study, Beijing, the most developed area in China, was taken as a case for deepening analysis about Asian cities. A time-series analysis on Beijing from 2014 to 2016 was conducted mainly to reveal more evidence on the association between air pollutants and their health effects on respiratory diseases. It should be noted that policies to decrease the concentration of air pollutants in Beijing, such as “Action Plan for Air Pollution Control” and relevant detailed plans, focused only on particulate matter, leaving gaseous emissions out of consideration. This study fills the research gap and focuses on gaseous pollutants and their health effects. 

## 2. Materials and Methods

### 2.1. Data

Beijing, the capital of China, covers an area of 1401 km^2^ and has approximately 21.7 million population [36], and it has been the most rapidly developing area in China. The daily outpatient number of respiratory diseases between 1 January 2014 and 31 December 2016 were collected from the respiratory diseases department of one of the central hospitals in Beijing and all the patients had been diagnosed with respiratory diseases by doctors. This Class-Three, Grade A level hospital shares its fame in treating respiratory diseases. Thus, it often receives patients from all parts of the city and is regarded as a representative hospital of respiratory diseases patients. Besides, this hospital is open to the public, and serves as one of the comprehensive centers for a range of integrated health services as well as education and scientific research. However, no available specific information of severity and structure of the patients, such as age-, sex- and disease-specific data, were included in our data. Daily concentration of six main air pollutants (O_3_, NO_2_, CO, SO_2_, PM_10_ and PM_2.5_) were collected from Beijing Municipal Environmental Protection Monitoring Center. Data from 12 fixed-site air quality monitoring stations throughout the city, including eight urban stations, three suburban stations, and one background station [37,38], were averaged to provide citywide daily estimates, and the missing values were filled by polynomial interpolation [39]. The issue of missing data is negligible because the missing proportion is quite small in all stations (<0.1%). The daily mean concentrations of air pollutants were calculated as the 24-h mean concentration for all pollutants except for O_3_. For O_3_, the 8-h mobile mean concentration was calculated as it reflects the greatest health-relevant exposure to O_3_ [40]. The air pollutants are measured in µg/m^3^ (how many micro-grams there are in one cubic meter of air), except for CO whose unit is mg/m^3^. To control for the effects of weather on number of outpatients, 24-h average meteorological data (daily mean temperature and relative humidity) were obtained from China Meteorological Data Sharing Service System.

### 2.2. Distributed Lag Non-Linear Model

Distributed lag non-linear models (DLNMs) represent a modeling framework to flexibly describe potentially non-linear and delayed effects in time-series data [41]. The delayed effects usually refer to the phenomenon that short-term health effects of environmental stressors, such as high levels of pollution, will last some days after its occurrence and patient’s exposure [42,43]. 

The conventional way to deal with delayed effects is to use distributed lag models (DLMs). However, this group of models seems to have difficulty in explaining the non-linear relationship. Although several extensions have already been proposed including applying piecewise function [44] or quadratic terms [45], their ability to explain dependency is still somewhat limited [46]. Thus, a useful extension that aims to capture the non-linear relationships both in the space of the predictor and along lags is developed, leading to the family of DLNM. In other words, DLNM relax the constraints of DLM to express non-linear relationship, which makes DLM a special case of DLNM. To achieve it, DLNM improves the function that is generally used to describe the smoothed relationships between the variables *x_t_* and the linear predictor $s\left(x_{t};\eta \right)$, defined by the parameter vectors η. The improved basic algebra of this function can be illustrated as follows

(1) $$s\left(x_{t};\eta \right)=\overset{v_{x}}{\underset{j=1}{\sum }}\overset{v_{l}}{\underset{k=1}{\sum }}r_{tj}^{T}C_{k}\eta_{k}=w_{t}^{T}\eta ,$$

where *r_tj_* is the vector of lagged exposures for the time *t* transformed through the basis function *j*. *C* is a basis matrix to describe the effects of lagged exposures. The vector *w_t_* is obtained by applying the cross-basis functions to *x_t_*. More details could be found in previous researches [46,47,48].

### 2.3. Statistical Analysis

The association between risks of respiratory diseases and air pollutants exposure was estimated by Quasi-Poisson models with covariates (temperature, relative humidity, time, the day of week) under the framework of distributed lag non-linear models, as the daily number of consultations was small and typically followed a Poisson distribution [49,50]. The DLNM lag range has been set from 0–8 days, since recent publications show little evidence of a strong association with patients’ hospital admissions at a lag of ≥3 days [51] and very seldom test the association with hospital admissions beyond 8 days. Following some previous studies [33,52,53], this study applied natural smooth functions (ns) of calendar time with 7 degrees of freedom (df) to control time trend, 6 df for temperature (lag 0) and 3 df for relative humidity (lag 0) respectively to adjust for the potential non-linear confounding effects of weather conditions. Day of the week was also included in the model as categorical variables.

In consideration of the possible combined health effects from gaseous pollutants and particulate matter, the effects of each gaseous pollutant were studied individually and in combination with particulate matters. Namely, PM_2.5_, PM_10_ were added into the gaseous model both separately and jointly at the same lag with gaseous pollutants through DLNM [54]. 

After establishing basic models, sensitivity analysis was conducted with respect to different parameter choices, as they can potentially have crucial impacts on the results of DLNMs. 

All data in the database were double-checked and the relevant statistical analysis was carried out with R software (version 3.4.3) (R Foundation for Statistical Computing, Vienna, Austria) using packages dlnm and splines. The results of all the statistical tests with *p* < 0.05 were considered significant. Unless specified, the results were presented in terms of Relative Risk (RR) indicator defining the relative increase in the number of outpatients per 10 µg/m^3^ additional increase of the air pollutant concentration. The 95% confidence intervals (CIs) of the estimates were also provided. CO results were presented with a measurement unit of mg/m^3^.

## 3. Results

Table 1 summarizes the basic statistics on outpatients of respiratory diseases, air pollution and weather data. From 1 January 2014 to 31 December 2016, a total of 307,484 outpatients with respiratory diseases were recorded. On average, there were approximately 276, 289 and 274 daily patients respectively in the years from 2014 to 2016. The number of outpatients was comparatively higher in cold season (October to March), which was 289 patients on daily average, compared to warm season (April to September), which was 271 patients on daily average.

As to air pollutants, there was a decreasing trend of concentration from 2014 to 2016. However, particulate matter remained at high level, as well as ozone and nitrogen dioxides. Pollutants concentration was higher in cold season, only ozone concentration was higher in warm season. Some publications show similar trends [38].

During the study period, meteorological conditions in Beijing were comparatively stable and consistent with the existing literature [55]. The mean temperature was 14.10 ± 13.32 °C and average relative humidity was 52.07%, ranging from 8% to 98%.

Pearson correlation coefficients between different air pollutants are presented in Table 2. Generally, NO_2_, CO, SO_2_, PM_2.5_ and PM_10_ exhibited strong positive correlation to each other, which can be explained by the fact that these pollutants mainly come from traffics [9] since Beijing no longer has heavy industries. However, ozone showed negative correlations with other pollutants, and the correlations were comparatively weaker.

**Figure 1:**
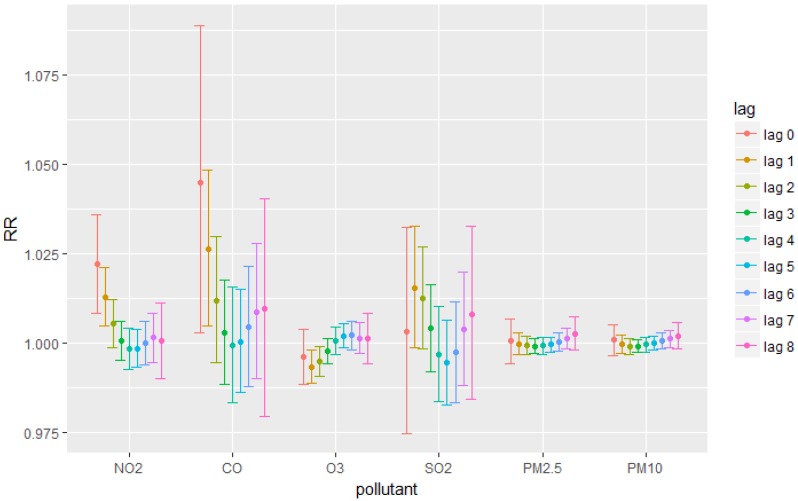
Relative Risks for each pollutant at each lag.

After adjusting for covariates including temperature, relative humidity, time and day of the week, the results measuring the RR were obtained with 95% CI. Results (Figure 1) show that gaseous pollutants, except for SO_2_, were more likely to have significant results than particulate pollutants. The concentration of CO and NO_2_ had positive correlation with respiratory diseases on the day of exposure (at lag 0 (1.045 (95% CI: 1.003, 1.089) for CO and 1.022 (95% CI: 1.008, 1.036) for NO_2_)) and on the first day after the exposure (lag 1 (1.026 (95% CI: 1.005, 1.048) for CO and 1.013 (95% CI: 1.005, 1.021) for NO_2_)). Ozone had insignificant relationship with respiratory diseases at most of the lags, except for significant negative results at lag 1 (RR = 0.993 (95% CI: 0.989, 0.998)) and lag 2 (RR = 0.995 (95% CI: 0.991, 0.999)). No significant results were observed for SO_2_ and particulate matters (PM_2.5_ and PM_10_).

**Figure 2:**
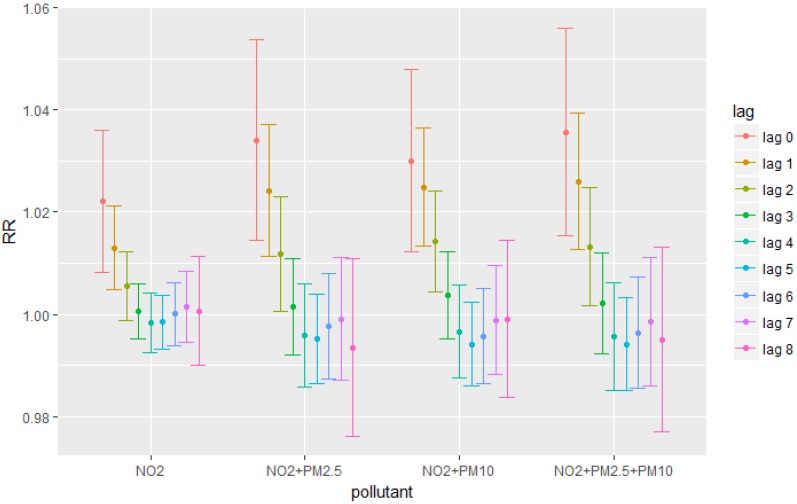
Relative Risks for different pollutants combination at each lag.

Figure 2 presents the model results combining the effects of particulate matters and NO_2_. The purpose is to show that unlike stated in several previous papers, our results indicate that gaseous pollutants in combination with particulate matters still had significant health effects. For example, results in Figure 2 showed that lag 2 was an extra significant lag for NO_2_ compared to the result without adding particulate matters. Since controlling for the effects of particulate matter almost did not change our single-pollutant results, to avoid collinearity, this paper mainly discussed about the results from single-pollutant models.

Sensitivity analysis shows that the RR estimates were robust to the degree of freedom in the smooth function of time adjusted for seasonal and long-term trends or for temperature (more details could be found in the Supplementary Materials, Figures S1 and S2). In other words, the results were not significantly influenced by changing parameters settings.

## 4. Discussion

Focusing on the effects of gaseous pollutants on outpatients of respiratory diseases, this study combined epidemiological and environmental research methods to investigate associations between short-term exposure to major air pollutants and the number of outpatients with respiratory diseases. This study was based on new evidence: the outpatient data were from a major and representative hospital for respiratory diseases in Beijing; and daily meteorological and monitor data of air pollutants were collected in the period from 2014–2016, which was a period after the Air Pollution Prevention and Control Action Plan adopted in Beijing in 2013.

Our study demonstrated that gaseous pollutants can produce rather significant health impact even during a short-term exposure. The observed health effects from CO exposure gradually decreased with the number of days from the day of exposure (from lag 0 to lag 4), and the effects remained significant in the first two days. This finding is supported by other studies [56,57,58]. The short-term significant positive effect of NO_2_ was also consistent with other existing studies [38,55,59,60,61]. The similar patterns of health effects from CO and NO_2_ exposure found in our paper may be because both pollutants are associated with vehicle exhaust [57]. As a matter of fact, all heavy industries had been moved out of Beijing, which made vehicle exhaust to the only one major emission source of air pollutants in Beijing [62]. Thus, the results of this study also suggested that CO, as well as NO_2_, are important components of the air pollution mixture in Beijing [38,57,63], and their acute adverse health effects should not be ignored.

Ozone showed significant short-term protective effect in our study [38,64]. The mechanism behind this finding should be investigated further. Some previous studies have proven that under certain circumstances, ozone can serve as a ‘protector’. For example, O_3_ in autohemotherapy has been used to treat ischemic disorders [65] and oxygen-ozone gaseous mixture is regarded as effective in recovering damaged brain tissues [66], improving cerebrovascular rheology and strengthening antioxidant in hypoxic brains [67]. Although these evidences might not be closely relevant to respiratory diseases, this paper used them as examples to show that they might give some inspiration about the protective effects of ozone. On the other hand, since the concentration of ozone was much higher in summer than in winter while outpatient numbers remain lower in summer than in winter, the association of concentration of ozone and outpatient numbers should be negative. Overall, the mechanism of ozone and its health effects need further studies.

Despite that some previous studies reported significant positive relationship between SO_2_ and respiratory diseases [55,68], no confirmation to these results was found in our data. This may be because much lower pollutants concentration data were investigated in this study than in those previous papers. For instance, mean concentration of SO_2_ calculated from our data equaled 18.11 µg/m^3^ with maximum of 133 µg/m^3^ in 2014 and 9.99 µg/m^3^ with maximum of 56 µg/m^3^ in 2016, which were consistent with the data in governmental report (http://www.xinhuanet.com/politics/2018-01/03/c_129781896.htm). Meanwhile, mean and maximum daily concentration of 44.05 µg/m^3^ and 1519.50 µg/m^3^ and of 67 µg/m^3^ and 383 µg/m^3^ were mentioned in in Zhang’s and Peters’ papers respectively. This potential explanation of the difference between studies suggest a need to consider the threshold that leads to the adverse health outcomes of SO_2_.

Although the association pattern of particulate matters in our studies were consistent with the existing literature [38,69], unlike most of the publications that reported significant positive association between particulate matter and respiratory diseases, no significant results were observed in our analysis [70,71]. This can be explained by the differences between the sources and structure of the data across the studies. The data in these studies can be characterized as highly heterogeneous due to a mix of patients who were in different age and social groups, and high variability to pollutants exposure. Despite the patient mix somewhat complicates our analysis, the patient mix is a common problem acknowledged in time-series studies related to environment and health, especially in studies on mortality. The unclear data composition sheds light on why no significant results were observed for particulate matter in our study. Two papers [38,51] reported different relationships between PMs and different type of respiratory diseases. For example, PM_2.5_ had significant positive association with upper respiratory tract infection (at lag 0, lag 4 and lag 5), and community-acquired pneumonia (at lag 0, lag 1 and lag 5), but no significant association with acute exacerbation of chronic obstructive pulmonary disease, acute exacerbation of asthma, and acute exacerbation of bronchiectasis [38]. As such, whether the results are significant, partly depends on the structure of the outpatients’ data. Wide variability of outpatients may also lead to wider confidence intervals, which heavily influence the significance of our results. Taking closer view on the non-significant results, the lower confidence interval was quite close to 1, for example, the lower confidence interval for PM_10_ was 0.99962 at lag 0 and 0.99968 at lag 1. If the variance of data were reduced, the results for particulate matter could become consistent and significant. This partly explains why our results on particulate matter showed similar patterns to other existing studies, but insignificant lag effects. Thus, the non-significant results of our studies related to particulate matters are reasonable. 

This study has many advantages. To our knowledge, this study was one of the few studies that used outpatient data, instead of hospital admission data or emergency room visits data, to examine the health effects of air pollutants. In Beijing, people with short-term infected phenomenon prefer outpatient departments over emergency rooms because of the following reasons. First is that most of the doctors serve during daytime at outpatient departments, and Chinese people generally prefer getting diagnosis from the established doctors no matter what or how severe the disease is. At the same time, unlike western countries, using outpatient department is often more convenient than using emergency rooms in China. Regarding the reliability of data and results, using hospital admission data may also be tricky since patients usually wait several days for a hospital bed, and this may cause some trouble in identifying delayed effects. Therefore, outpatient departments receive the vast majority of patients with respiratory diseases. As a result, using data from outpatient departments may be the best option to investigate the lag distribution of short-term health effects of ambient pollutants.

From the view point of data and method, our newly collected data revealed some new findings compared to previous studies. Data monitored from 2014 to 2016 on air pollutants and health conditions were used, while most studies on Beijing air pollution used data before 2013. Surprisingly, the effects of gaseous pollutants remain significant even after controlling for particulate matter in this study. In addition, carefully examined robust results from sensitivity analysis made us confident in identifying the difference between our studies and other existing research. If taking time effect of the data into consideration, it is very likely that this study, compared with earlier studies, identified that the element composition and the sources of particulate matter in Beijing air have changed because of the adopted air pollution regulations. Thus, the effects of particulate matter dropped while the effects of gaseous pollutants increased, which also showed that gaseous pollutants might be a major concern for Beijing in the near future. Compared to the current policy, which only focused on controlling particulate matter concentration, more measures should be taken to prevent or eliminate the negative health effects of gaseous pollutants.

Beijing is one of the most developed areas in a fast-developing country. Results of this study for Beijing are quite general and may also apply to other citizens in developing areas or developed area in developing countries, where a growing number of people living under the risk of being affected by respiratory diseases.

To be noted, any inference based on this paper for policymaking should be careful. As unavailability of detailed data (e.g., age- and sex-specific data) is a major limitation of this study, potential bias of this study should be taken into consideration. More specifically, as the studied hospital is especially famous for its treatment of bronchial asthma, interventional respiratory disease, and pulmonary interstitial disease, it might be reasonable to assume that the data in this study consisted mainly of outpatients with the above lower respiratory diseases. As to the severity of these diseases, this study assumed that most of the patients of the outpatient department had mild or moderate symptoms, as patients with severe symptoms would usually go to the emergency department and the hospital admission department instead.

## 5. Conclusions

This study concluded that short-term exposure to gaseous pollutants was harmful for humans. Also, this study emphasized that the health effects of air pollutants in Beijing have changed, and public health policy should pay special attention to gaseous ones. For air pollution control measures, closing unqualified industries and relocating heavy industry factories is not the only dimension. Controlling vehicle-emitted pollutants must be properly addressed to minimize the pollution impact on health. Regarding health regulations, adjustments of medical sources distribution accordingly require careful consideration. Beijing is a highly urbanized area in a rapidly developing country. Thus, our study can be useful not only to Beijing, but also to other mega cities in developing countries, in helping their sustainable development in the future.

## Figures and Tables

**Table 1:** Summary statistics of daily outpatients, air pollutant concentrations, and weather conditions, 2014–2016.

Variables	2014	2015	2016
Number of outpatients	275.96 ± 159.70	288.80 ± 163.53	273.59 ± 149.11
Air Pollutant Concentrations			
CO (mg/m^3^)	1.21 ± 0.83	1.30 ± 1.12	1.16 ± 0.96
NO_2_ (µg/m^3^)	53.72 ± 23.30	49.02 ± 24.55	48.21 ± 24.29
O_3_ (µg/m^3^)	104.52 ± 64.23	99.51 ± 65.88	95.76 ± 65.20
SO_2_ (µg/m^3^)	18.11 ± 21.14	12.76 ± 13.78	9.99 ± 10.30
PM_2.5_ (µg/m^3^)	83.80 ± 68.80	80.11 ± 71.90	72.89 ± 63.51
PM_10_ (µg/m^3^)	115.86 ± 74.58	100.61 ± 84.26	97.01 ± 73.73
Meteorological Measures (24 h average)			
Temperature (°C)	15.39 ± 15.52	13.36 ± 10.29	13.64 ± 13.71
Relative Humidity (%)	51.00 ± 18.98	54.24 ± 20.16	50.92 ± 20.20

**Table 2:** Correlation between the pollutants.

Variables	CO	NO_2_	O_3_	SO_2_	PM_2.5_	PM_10_
CO	1.000	-	-	-	-	-
NO_2_	0.815 *	1.000	-	-	-	-
O_3_	−0.383 *	−0.388 *	1.000	-	-	-
SO_2_	0.603 *	0.629 *	−0.323 *	1.000	-	-
PM_2.5_	0.829 *	0.793 *	−0.136 *	0.533 *	1.000	-
PM_10_	0.707 *	0.748 *	−0.090 *	0.525 *	0.840 *	1.000

* significant difference (*p* < 0.05).

## Supplementary Materials

The following are available online at http://www.mdpi.com/1660-4601/15/9/1969/s1, Figure S1: Relative Risks with 10 µg/m^3^ increase of NO_2_ by degrees of freedom of time trends, Figure S2: Relative Risks with 10 µg/m^3^ increase of NO_2_ by degrees of freedom of temperature.
