# Supplementary material for: Health Impacts of Exposure to Gaseous Pollutants and Particulate Matter in Beijing—A Non-Linear Analysis Based on the New Evidence
Source: Int J Environ Res Public Health. 2018 Sep 10;15(9):1969. doi: 10.3390/ijerph15091969 (PMC6165060; doi:10.3390/ijerph15091969)
Supplement: Supplementary file 1 [file ijerph-15-01969-s001.pdf]

Supplementary materials for sensitivity analysis

Figure 1 and Figure 2 present the results from a single-pollutant model for NO_2 at lag 1 as an example, since other lags generated similar trends. The results for other pollutants were also robust with respect to different variable settings, i.e. degrees of freedom, time and temperature trends adjustments, etc.

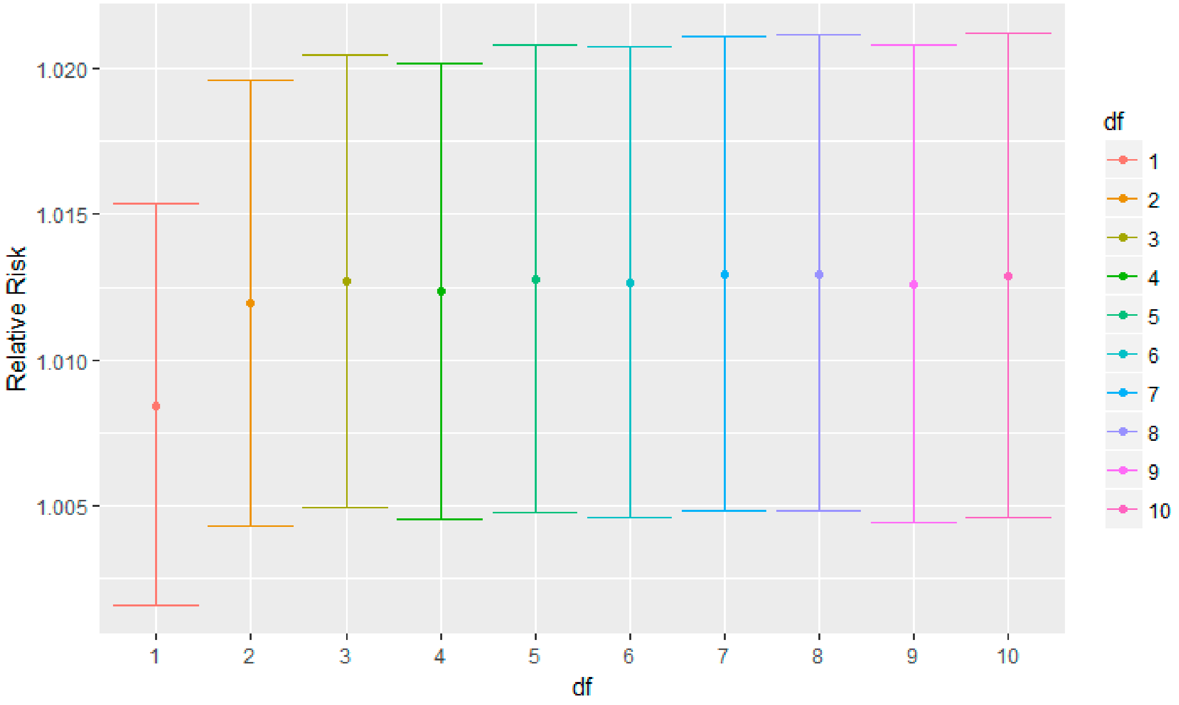

Figure S1. Relative Risks with 10 μg/m³ increase of NO_2 by degrees of freedom of time trends.

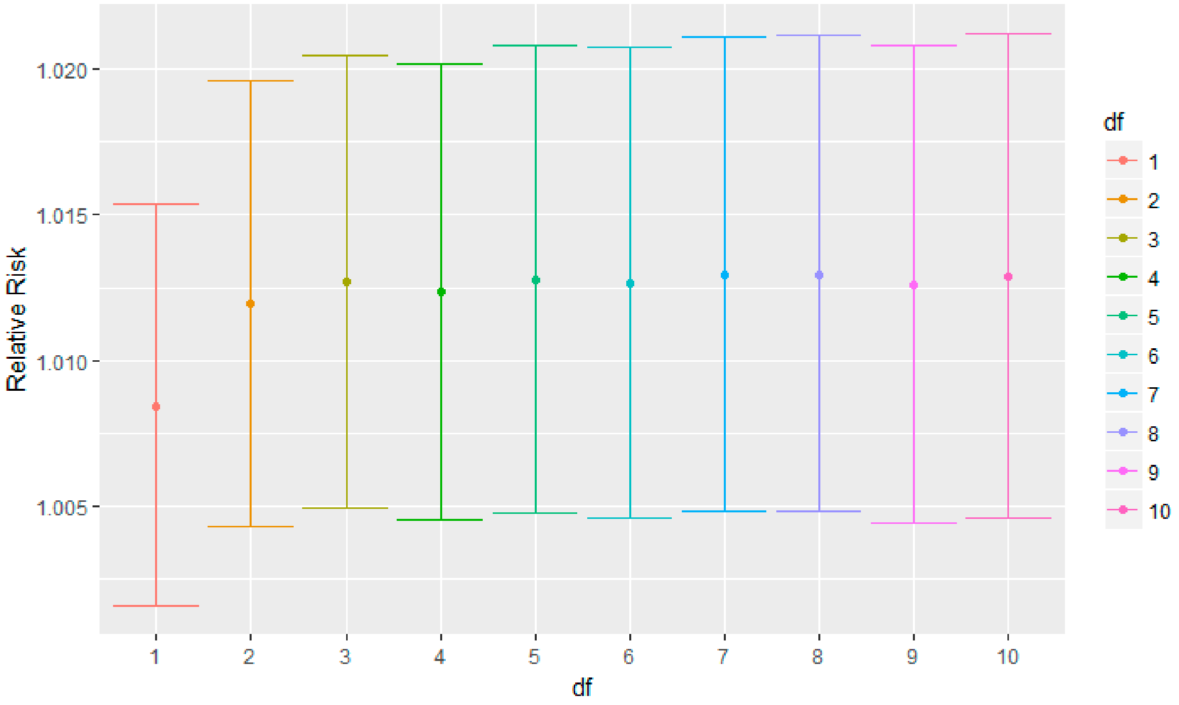

Figure S2. Relative Risks with 10 μg/m³ increase of NO_2 by degrees of freedom of temperature.
